# Combined life-threatening thromboses and hemorrhages in a patient with afibrinogenemia and antithrombin deficiency

**DOI:** 10.1186/s12959-018-0162-8

**Published:** 2018-04-04

**Authors:** S. Le Quellec, A. Desjonqueres, L. Rugeri, H. Desmurs Clavel, F. Farhat, L. Mechtouff, Y. Dargaud

**Affiliations:** 1 Unité d’Hémostase Clinique, Hôpital Cardiologique Louis Pradel, 28, avenue Doyen J. Lepine, Bron, F-69500 Lyon, France; 2 Service de Médecine Interne, Hôpital Edouard Herriot, Lyon, France; 3 Service de Chirurgie Cardiaque, Hôpital Cardiologique Louis Pradel, Lyon, France; 4 Service Neuro-Vasculaire, Hôpital Neurologique Wertheimer, Lyon, France

**Keywords:** Aibrinogenemia, Antithrombin deficiency, Myocardial infarction, Stroke, Pulmonary embolism, Bleeding

## Abstract

**Background:**

Patients with congenital afibrinogenemia suffer from spontaneous recurrent severe bleeding. While fibrinogen concentrates are known to effectively treat bleeding episodes, thrombotic complications often occur upon replacement therapy, rendering clinical management highly challenging.

**Case Presentation:**

We hereby report a case of combined afibrinogenemia and congenital antithrombin deficiency manifested by recurrent life-threatening bleeding, as well as spontaneous severe arterial occlusion, such as acute coronary syndrome and stroke, and venous thromboses like pulmonary embolism.

Secondary fibrinogen prophylaxis is recommended following any initial life-threatening bleeding episode in patients with afibrinogenemia, yet the high associated risk of thrombosis illustrates the complexity of choosing the most effective prophylaxis strategy combining fibrinogen concentrate with antithrombotic agent for optimal protection against the risk of both severe bleeding and thrombosis. For our patient, the thrombin generation assay objectively confirmed her prothrombotic tendency.

**Conclusion:**

This case may help us better understand the pathophysiology of arterial thrombosis in afibrinogenemia, while highlighting the difficulty of managing such complications.

## Background

Congenital afibrinogenemia is a rare bleeding disorder with an estimated prevalence of 1:1000 000 [1]. The main symptom of this condition characterized by complete absence of circulating fibrinogen is bleeding, which can affect all tissues including the umbilical cord at birth, mucocutaneous zones, joints, soft tissues, genito-urinary tract, and central nervous system. Poor wound healing, spontaneous splenic rupture, and bone cysts have also been described in this context. In addition to bleeding symptoms, afibrinogenemia can be associated with rare venous or arterial thrombotic events [2]. Bleeding episodes are typically treated with fibrinogen concentrates and antifibrinolytic agents. For severe bleedings, current guidelines recommend target levels of fibrinogen activity >1.0 g/L [3]. In patients with afibrinogenemia, the absence of inactivation by fibrin is associated with increased free thrombin activity compared to healthy controls [4]. Fibrinogen replacement therapy can therefore increase the risk of thrombotic complications [5–7], thus rendering the management of bleeding episodes most challenging. Extreme care needs to be taken with replacement therapy in these patients, consisting of careful monitoring of plasma fibrinogen activity. This case report describes a very rare case of combined afibrinogenemia and antithrombin deficiency with major thrombosis risk, emphasizing the need for careful medical evaluation of patients with afibrinogenemia that should not only focus on treating the bleeding episodes.

## Case Presentation

A 49-year-old non-smoker female with known afibrinogenemia and antithrombin (AT) deficiency was admitted to the emergency room (ER) for acute substernal chest pain. She had a history of pyelonephritis, had received treatment for a multinodular goiter that had caused hypothyroidism, and also had congenital deafness. Her parents were consanguineous, and her father died from a surgically-induced pulmonary embolism. Her brother suffered from the same combined afibrinogenemia and AT deficiency. Her sister died from pulmonary embolism during her first pregnancy, at the age of 22, and had also suffered from AT deficiency yet with normal fibrinogen levels.

Afibrinogenemia was diagnosed when the patient was 3 months old, following a trauma-induced intracranial hemorrhage. DNA sequencing, performed several years after the diagnosis, highlighted a homozygous (c.510 + 1G > T) substitution in the splicing site of the 4th intron of the fibrinogen alpha-chain. Routine coagulation test results revealed dramatically-prolonged activated partial thromboplastin time (aPTT) (>240 s) and a prothrombin time (PT) >120 s, with undetectable levels of either fibrinogen activity or antigen <0.1 g/L, and normal platelet count of 240.10 [8]/L. Thrombin time and reptilase time were also significantly prolonged (>120 s; normal values below 20 s).

The patient had a history of several severe thromboses and bleeding episodes. Her bleeding history started with intracranial bleeding induced by severe head trauma suffered at 3 months old, which was followed by several trauma-induced hematomas, spontaneous meningeal hemorrhage at 11 years old, intra-alveolar pulmonary hemorrhage at 23, menorrhagia, two episodes of hemoperitoneum induced by ruptured ovarian cysts, six first-trimester miscarriages due to severe hemorrhagic complications, and acute unprovoked cerebellar hemorrhage at 30 years old. Most of the bleeding episodes that occurred during her childhood and youth were treated with cryoprecipitate and fresh frozen plasma. She was shown to be hepatitis C antibody- and RNA-positive, with normal liver enzyme levels. The patient was taking several supplements for iron deficiency anemia due to heavy menstrual blood loss, and refused prophylaxis with fibrinogen concentrates, as they require regular intravenous infusions. Moreover, she was working in the traveling family circus with a lifestyle of “perpetual traveler”, rendering clinical follow-up very difficult.

**Fig. 1:**
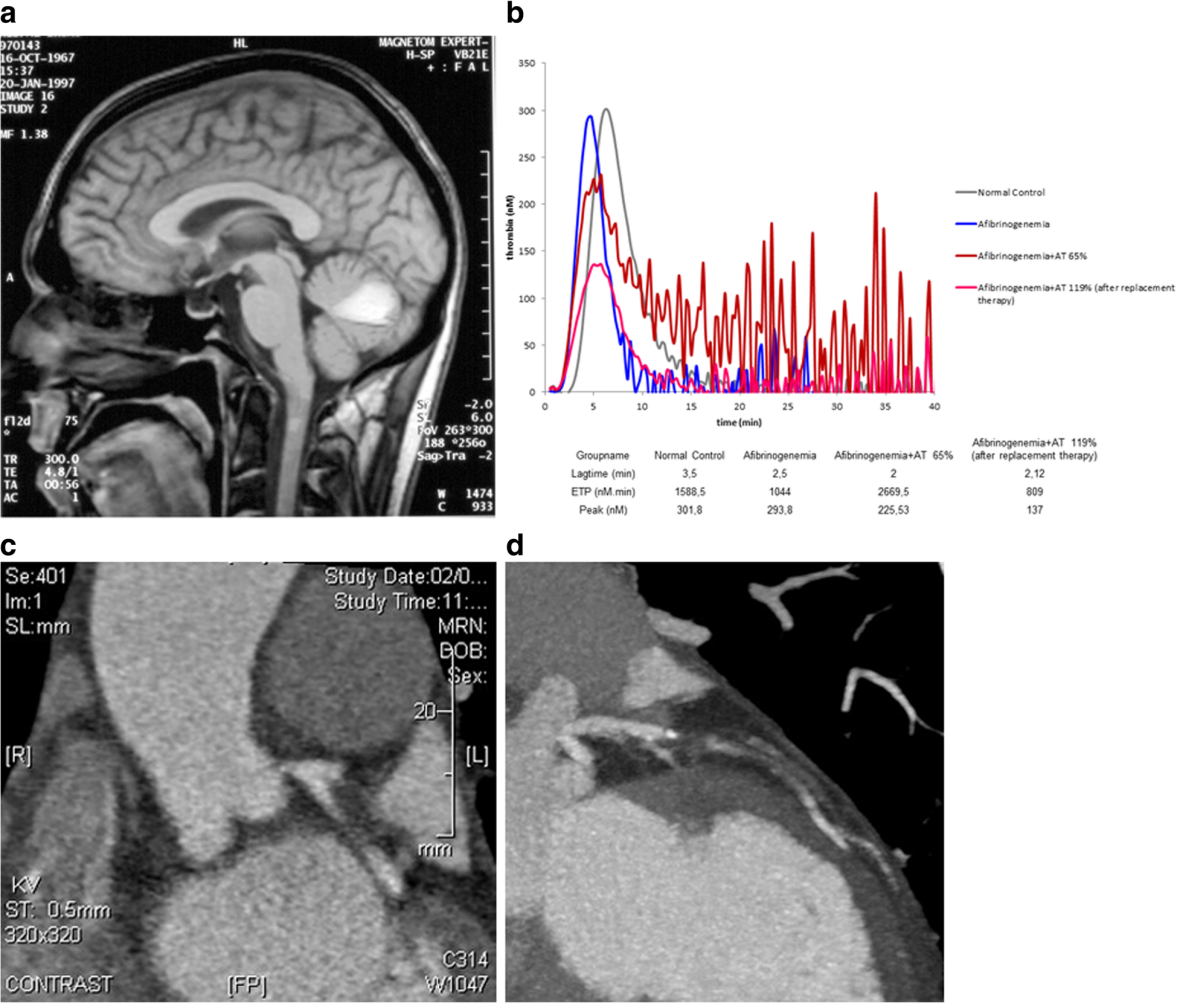
**a** Sagittal computed tomography angiography showing a left cerebellar hemisphere hemorrhage. **b** Thrombin generation curves obtained with 5pM tissue factor and 4 μM phospholipids (final concentration) in platelet-poor plasma using calibrated automated thrombin generation assay (Stago, Asnières, France). The area under the thrombin generation curve (or endogenous thrombin potential) is significantly higher in the patient (red) compared to another subject with afibrinogenemia (blue) or a representative normal control (grey). In this patient with combined inherited antithrombin and fibrinogen deficiency, increased thrombin generation is due to insufficient inhibition of thrombin. Thrombin generation is decreased after infusion of 30 U/kg antithrombin concentrate (pink). **c** Visualization of the left coronary artery with computed tomography coronary angiogram showing 80% stenosis in the common trunk. **d** Visualization of the left coronary artery with computed tomography coronary angiogram showing 50% stenosis in the anterior interventricular branch of left coronary artery

At the age of 30, she exhibited a spontaneous cerebellar hemorrhage (Fig. 1a) and received her first replacement therapy with fibrinogen concentrates, after which she developed a proximal deep vein thrombosis (DVT) complicated by pulmonary embolism (PE) four days after the introduction of fibrinogen concentrate, while her plasma fibrinogen level was 1.2 g/L. The fibrinogen infusions were immediately stopped, and unfractionated heparin (UFH) was initiated. When her fibrinogen level dropped to <0.5 g/L, 24 h following withdrawal of the fibrinogen concentrate, fibrinogen replacement therapy was reinitiated, with a target plasma fibrinogen of 0.5–0.8 g/L, and UFH was concomitantly administered for a total duration of 6 weeks. No bleeding complications occurred, and her cerebellar bleeding was not aggravated during UFH therapy. The patient fully recovered from this episode.

Two years later, at 32 years, she presented an acute coronary syndrome (ACS) with an ulcerated atheromatous plaque in the left anterior descending coronary artery. However, due to there being only a limited perfusion defect and no occlusion on coronary angiography, reperfusion therapy was not initiated given her high risk of bleeding. She had no cardiovascular risk factors, except for slightly-elevated cholesterol levels. A combined treatment of low molecular weight heparin (LMWH) and fibrinogen was prescribed. On replacement therapy, the patient exhibited bilateral calf DVT, which was treated with LMWH until complete resolution of the venous clots, achieved in 10 days. Following hospital discharge, the patient pursued a treatment at home consisting of 40 mg atorvastatin per day, with a target LDL cholesterol level < 1 g/L. No aspirin was prescribed for this patient due to her high bleeding risk. As several thromboses were reported in the patient’s family, and given the known AT deficiency of her sister, thrombophilia testing was then performed, revealing heterozygous antithrombin (AT) deficiency at 65% by means of chromogenic assay (Biophen AT anti-(h)-Xa LRT, Hyphen BioMed, Neuville sur Oise, France). The reference AT in adults ranges between 80 and 120%. Genetic sequencing revealed a point mutation L131F in the 2nd exon of the AT gene. As a result, this proved to be a very rare case of combined afibrinogenemia and AT deficiency. Plasma AT was therefore measured in her brother, confirming similar combined inherited AT and fibrinogen deficiencies.

Thrombin generation was measured using Calibrated automated thrombin generation assay (Stago, les Asnières, France) in the presence of a low concentration of tissue factor 1pM, in accordance with the recent recommendations of the ISTH [9]. Thrombin generation (TG) assay revealed the patent had a higher TG capacity compared to other afibrinogenemia patients (Fig. 1b). The area under the thrombin generation curve (ETP) representing the enzymatic activity of thrombin was very high (ETP = 2670 nM.min) in the patient, in accordance with her clinical history of thrombosis. AT deficiency was probably the main reason of the increased thrombin generation capacity in our patient. A slight TG decrease was observed following infusion of AT concentrate at 30 U/Kg (Fig. 1b). In light of these clinical and laboratory findings, we decided to normalize the patient’s AT level prior to any fibrinogen concentrate infusion, in an effort to reduce the risk of thrombosis related to replacement therapy.

At 49 years old, the patient consulted at the ER for severe acute chest pain. Her blood pressure on admission was 87/57 mmHg, her heart rate 87 bpm, and her physical examination unremarkable. Upon admission, laboratory tests revealed increased alanine aminotransferase activity (50 U/L [normal: 3–26 U/L]) and aspartate aminotransferase (121 U/L [normal: 6–18 U/L). Her troponin-T level was dramatically increased (24,693 ng/L [normal: <20 ng/L]). Her C-reactive protein was 62.6 mg/L (normal <5 mg/lL). Routine coagulation tests showed activated partial thromboplastin time (aPTT) >240 s and prothrombin time (PT) >120 s. The patient’s plasma fibrinogen level was undetectable for both activity and antigen assays. The electrocardiogram (ECG) was normal. Non-ST-elevated myocardial infarction (NSTEMI) was suspected. Echocardiography confirmed the diagnosis and revealed an impaired ejection fraction of 45%, with akinesia in the anteroseptal, anteromedial, and apical segments. The CT coronary angiogram revealed 80% stenosis in the common trunk of the left coronary artery and 50% stenosis in the anterior interventricular branch of the left coronary artery (Fig. 1c,d). The right coronary artery and circumflex coronary artery were normal. A diagnosis of NSTEMI was established, and double coronary artery bypass surgery was scheduled.

The surgery was performed, following replacement therapy with 30 U/Kg antithrombin concentrate (Aclotine®, LFB-Biomedicaments, Les Ulis, France) and 1.5 g fibrinogen (Clottafact®, LFB-Biomedicaments), along with UFH at the typical coronary bypass dosage. The surgery was successful, whilst carefully controlling the combined anticoagulant and procoagulant molecules. No excessive bleeding occurred. UFH was maintained for 3 weeks following surgery. Antithrombin and fibrinogen infusions were calculated following laboratory results with the aim of maintaining plasma AT >80% and fibrinogen between 0.5 and 1 g/L. All pro- and anti-coagulant treatments were discontinued 2 days before hospital discharge, on post-op Day 23. Two days after treatment withdrawal, the patient developed a segmental PE treated with LMWH in association with antithrombin and fibrinogen concentrates for 2 months, as described above.

In this patient who had suffered two acute coronary heart attacks, secondary prophylaxis with 75 mg aspirin/day was recommended, combined with antithrombin and fibrinogen infusions administered at least once a week, in order to keep plasma fibrinogen >0.5 g/L. However, the patient refused fibrinogen and AT prophylaxis, for his would require weekly injections. Therefore, antithrombotic prophylaxis with antiplatelet therapy (APT) was not initiated.

Five months later, the patient was admitted to the ER for spontaneous left upper limb monoplegia associated with left-sided facial paralysis, dysarthria, and left homonymous hemianopia. Cerebral computed tomography (CT) revealed multiple hypodense ischemic lesions in the right frontal and parietal lobes associated with a thrombus in the right internal carotid artery. Echocardiogram, Holter ECG, and supra-aortic branch evaluation using Doppler ultrasonography disclosed no additional lesions indicative of stroke. On admission, plasma fibrinogen was <0.1 g/L, and daily aspirin at 75 mg was initiated. No fibrinogen replacement therapy was prescribed, given her several previous VTE episodes following fibrinogen concentrate infusions. The follow-up CT, performed one week after starting aspirin, revealed an acute subdural hematoma (SDH) measuring 27 mm, probably induced by aspirin. As the patient was asymptomatic, aspirin was immediately discontinued, and she was administered antithrombin and fibrinogen concentrates twice a week for 4 months, resulting in significant regression of the SDH to 10 mm in its largest diameter. Eventually, she accepted long-term antithrombin and fibrinogen prophylaxis based on weekly infusions (30 U/Kg and 1.5 g, respectively), rendering anti-thrombotic prophylaxis with aspirin again possible, which was very carefully reintroduced at low doses of 75 mg, arbitrarily, administered every 3 days. Under prophylaxis, her fibrinogen trough levels were ≥0.3 g/L. The patient has not reported any breakthrough bleeds with the current prophylactic regimen. No recent thrombosis has occurred and follow-up cerebral CTs have shown that the SDH is remaining stable.

## Discussion

In patients with afibrinogenemia, venous thrombosis proves to be a common complication of the replacement therapy using fibrinogen concentrates, while arterial thromboses primarily manifest unprovoked in patients who have no risk factors for coronary artery disease [8]. The mechanism of this acute arterial occlusive disease in young adults is not fully understood. Some studies have highlighted the potential implications of increased thrombin generation (TG) [4, 10]. Thrombin acts as a powerful modulator of vascular tone, as well as migration and proliferation of vascular smooth-muscle cells, along with recruitment of macrophages into the atherosclerotic plaques. It also induces a pro- inflammatory response by activating G-protein-coupled-PAR receptors on various cells, including endothelial cells and platelets [11]. In addition, studies in transgenic mice models have indicated that the deletion of thrombin inhibitor heparin co-factor II (HC-II) promotes atherosclerosis [12]. The inhibition of thrombin with melagatran in apolipoprotein-E deficient mice reduces plaque growth and improves the stability of advanced atherosclerotic plaques [13]. Physiologically-generated thrombin is partly captured by fibrinogen, thrombomodulin, and other receptors, while free thrombin is rapidly inactivated by several inhibitors like antithrombin and HC-II. During atherosclerosis, a significant decrease in thrombomodulin on the endothelium has been reported [14]. This likely impairs the anticoagulant action of thrombin in atherosclerotic vessels, whilst further enhancing TG. Taken together in our patient with AT Budapest-3 mutation [15], highly increased TG causing excessive free thrombin in the plasma, not absorbed by fibrin, could have promoted atherosclerotic plaque development, rupture, and thrombosis.

The treatment of thrombotic events should be tailored to each individual case, with the objective of preventing thrombosis extension, as well as the risk of bleeding. There are currently no recommendations for long-term management of coronary disease in patients with afibrinogenemia [16]. Previously-reported cases of thromboses in patients with afibrinogenemia have demonstrated that arterial thromboses mostly occur in young patients, in the absence of traditional risk factors for atherosclerosis, suggesting that the pathophysiology of arterial events might differ in the afibrinogenemia context. Owing to the absence of fibrinogen, we can hypothesize that platelets may have played a major role in the pathophysiology of our patient’s arterial thromboses. Following revascularization surgery, APT and warfarin are typically prescribed in the general population [17, 18]. The bleeding risk related to this therapy type is theoretically high in patients with afibrinogenemia. The data on antiplatelet therapy in these patients prove to be scarce, although clopidogrel and aspirin have been safely prescribed in several patients without concomitant prophylaxis with fibrinogen concentrates [19, 20]. In our patient, with regard to her bleeding history, APT was recommended in association with fibrinogen prophylaxis, which she initially refused. The occurrence of SDH, which complicated the daily aspirin therapy, confirmed that such a treatment could not be safely considered without fibrinogen prophylaxis. Moreover, the occurrence of spontaneous ischemic stroke in the absence of thromboprophylaxis suggests the need for effective secondary antithrombotic prophylaxis, even in patients with severe coagulation disorders. We have yet to clearly define the optimal management of patients with afibrinogenemia and thromboembolic complications. Different approaches, ranging from supportive therapy to vasodilators, anticoagulants, antiplatelet agents or thrombolytics, have been reported [21]. In addition, it could be speculated that direct thrombin inhibitors like dabigatran could be effective in these patients who exhibit high free thrombin levels in the plasma. Nevertheless, thromboembolic complications are difficult to manage in patients with afibrinogenemia, given that it is crucial to cautiously administer anticoagulants whilst simultaneously administering fibrinogen preparations [5, 22, 23]. The use of anticoagulant agents was previously reported in patients with hypo- and/or dysfibrinogenemia with low levels of fibrinogen. However, the patient presented here has combined AT deficiency inducing significantly increased risk of thrombosis. Thus, in this particular case, after establishing the AT-deficiency diagnosis, it was decided to administer fibrinogen concentrates only once AT-deficiency had been corrected. We hypothesized that high amounts of thrombin present in the patient’s plasma could be partly neutralized by antithrombin, which would limit the risk of thrombosis related to replacement therapy. The patient has presented no further venous thromboembolisms concomitant to fibrinogen infusions. This is a very rare case of combined inherited deficiencies, in which AT deficiency was shown responsible for higher thrombin generation capacity compared to other cases with afibrinogenemia. Although these results cannot be extrapolated to all patients with afibrinogenemia, it is worth noting that some physicians do combine small doses of heparin with fibrinogen infusions [24] and this in an effort to prevent the risk of thrombosis upon fibrinogen replacement therapy.

## Conclusion

We herein report a rare case of combined inherited afibrinogenemia and AT-deficiency that may help us better understand the pathophysiology of arterial thrombosis in afibrinogenemia, whilst highlighting the difficulty of managing such complications.

## Abbreviations

APT: Antiplatelet therapy
aPTT: Activated partial thromboplastin time
AT: Antithrombin
DVT: Deep vein thrombosis
ECG: Electrocardiogram
ER: Emergency room
HC-II: Heparin co-factor II
LMWH: Low molecular weight heparin
NSTEMI: Non-ST-elevated myocardial infarction
PE: Pulmonary embolism
PT: Prothrombin time
SDH: Subdural hematoma
TG: Thrombin generation
UFH: Unfractionated heparin
